# Functional characterization of salicylate hydroxylase from the fungal endophyte *Epichloë festucae*

**DOI:** 10.1038/srep10939

**Published:** 2015-06-09

**Authors:** Karen V. Ambrose, Zipeng Tian, Yifei Wang, Jordan Smith, Gerben Zylstra, Bingru Huang, Faith C. Belanger

**Affiliations:** 1 Department of Plant Biology and Pathology, Rutgers University, New Brunswick, New Jersey 08901; 2 Department of Biochemistry and Microbiology, Rutgers University, New Brunswick, New Jersey 08901.

## Abstract

*Epichloë* spp. are symbiotic fungal endophytes of many cool season grasses. The presence of the fungal endophytes often confers insect, drought, and disease tolerance to the host grasses. The presence of the fungal endophytes within the host plants does not elicit host defense responses. The molecular basis for this phenomenon is not known. *Epichloë festucae*, the endophyte of *Festuca rubra*, expresses a salicylate hydroxylase similar to NahG from the bacterium *Pseudomonas putida*. Few fungal salicylate hydroxylase enzymes have been reported. The *in planta* expression of an endophyte salicylate hydroxylase raised the possibility that degradation of plant-produced salicylic acid is a factor in the mechanism of how the endophyte avoids eliciting host plant defenses. Here we report the characterization of the *E. festucae* salicylate hydroxylase, designated *Efe-shyA*. Although the fungal enzyme has the expected activity, based on salicylic acid levels in endophyte-free and endophyte-infected plants it is unlikely that expression of the endophyte salicylate hydroxylase is a factor in the lack of a host defense response to the presence of the fungal endophyte.

Many cool season grasses have symbiotic associations with endophytic fungi of the genus *Epichloë*^1^. These associations often benefit the host grass by providing increased insect, drought, and disease tolerance^2^,^3^,^4^. The insect tolerance is known to be due to the production of various alkaloids by the fungus^5^,^6^, but the underlying mechanisms of increased drought and disease tolerance are not as well established.

Recently we reported a quantitative comparative transcriptome analysis of *E. festucae*-infected versus endophyte-free *Festuca rubra* (strong creeping red fescue) using the high throughput sequencing approach of SOLiD-SAGE^7^. Among the abundant fungal transcripts was one whose best match to a functionally characterized gene was to a salicylate hydroxylase (salicylate 1-monooxygenase) gene (*nahG*) from the bacterium *Pseudomonas putida*. In *P. putida, nahG* is part of a naphthalene catabolic operon through which environmental naphthalene is converted to pyruvate and acetaldehyde^8^. Salicylic acid is produced as an intermediate in the degradation of naphthalene and is converted by NahG to catechol by removal of the carboxyl group at position 1 and introduction of a hydroxyl group at that position (Fig. 1). Similar salicylate hydroxylase genes have also been reported from other soil bacteria^9^,^10^.

Fungal salicylate hydroxylase enzymes have rarely been reported. Fungal salicylate hydroxylase enzymatic activities have been studied in the soil yeast *Trichosporon cutaneum*^11^, *Aspergillus nidulans*^12^, *Fusarium* spp.^13^ and *T. moniliiforme*^14^. In a recent study of the smut fungus *Ustilago maydis*, three putative salicylate hydroxylase genes were identified that had similarity to *Pseudomonas* spp. *nahG*^15^. One of the three candidate genes had the ability to utilize salicylic acid as a substrate, and hence was considered to be an active enzyme that was subsequently named Shy1 (Salicylate hydroxylase 1). To date, the *U. maydis* Shy1 is the only heterologously expressed, and functionally characterized fungal salicylate hydroxylase.

The plant hormone salicylic acid is known to be a critical factor in activation of plant defenses in response to pathogen attack^16^,^17^. Because of the importance of salicylic acid in plant disease resistance, it has been the topic of considerable research and much is known about the complex network of proteins involved in salicylic acid-mediated defense signaling, particularly in the model plant *Arabidopsis thaliana*^16^. One of the early lines of evidence that salicylic acid was a critical factor in plant defense signaling was through analyses of transgenic plants expressing the *P. putida nahG*, which oxidizes salicylic acid to catechol^18^,^19^. The *nahG*-expressing plants had reduced levels of salicylic acid and increased susceptibility to pathogen attack. It is well known that plants infected with the *Epichloë* endophytes do not exhibit hypersensitive responses commonly associated with pathogenesis^20^. The *in planta* expression of a putative endophyte salicylate hydroxylase raised the possibility that degradation of plant-produced salicylic acid is a factor in the mechanism of endophyte evasion of host plant defenses.

Here we report the functional characterization of the putative salicylate hydroxylase from *E. festucae*. The recombinant *E. festucae* enzyme did have salicylate hydroxylase activity. However, the *in planta* levels of salicylic acid do not support the hypothesis that fungal degradation of host produced salicylic acid is a factor in endophyte-infection not eliciting host plant defenses.

## Results

### Expression of *Epichloë festucae* Salicylate Hydroxylase

**Figure 2:**
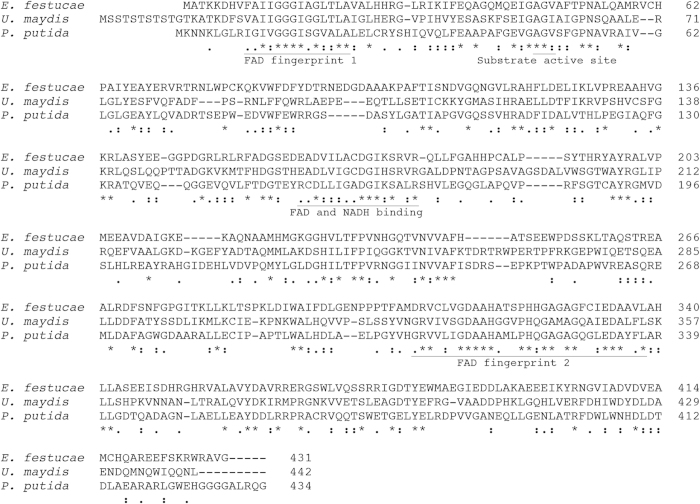
Comparison of deduced salicylate hydroxylase amino acid sequences from *Epichloë festucae* (accession KM400586), *Ustilago maydis* (accession XM_756284), and *Pseudomonas putida* (accession J05317). The conserved functional domains are underlined. An asterisk indicates identical residues in all sequences, a “:” indicates strongly conserved residues (score > 0.5), and a “ . ” indicates weaker conserved residues (score < 0.5) (Thompson *et al*., 1997).

The previous recovery of SOLiD-SAGE tags that mapped to an *E. festucae* EST sequence whose best Blastx match to a functionally characterized protein was to salicylate hydroxylase (NahG) from *P. putida* supports the expression of the putative salicylate hydroxylase gene^7^. The SOLiD-SAGE tags were recovered at 0.02% of the *in planta* expressed fungal tags. The corresponding gene in the genome sequence of the *E. festucae* 2368 isolate (http://www.endophyte.uky.edu/) is annotated as salicylate hydroxylase. However, the overall amino acid sequence identity between NahG and the *E. festucae* sequence is only 35%. Recently a *Ustilago maydis* salicylate hydroxylase, designated Shy1, has been functionally characterized^15^. The overall amino acid sequence identity of the *E. festucae* gene to the *U. maydis* salicylate hydroxylase is also low, at 36%. A comparison of the *E. festucae* putative salicylate hydroxylase amino acid sequence with the *P. putida* NahG and *U. maydis* Shy1 sequences is shown in Fig. 2. The bacterial salicylate hydroxylases are flavoprotein hydroxylases and the FAD and NADH binding sites have been identified^21^. These regions in the *E. festucae* putative salicylate hydroxylase and the *U. maydis* Shy1 are well conserved, relative to NahG (Fig. 2). The proposed substrate active site of the bacterial salicylate hydroxylases^10^ is also conserved in the fungal sequences. Based on the activity assays described below for the *E. festucae* protein, the previous *U. maydis* gene designation^15^, and on nomenclature recommendations for *Epichloë*^22^, the *E. festucae* gene is designated *Efe-shyA*.

**Figure 3:**
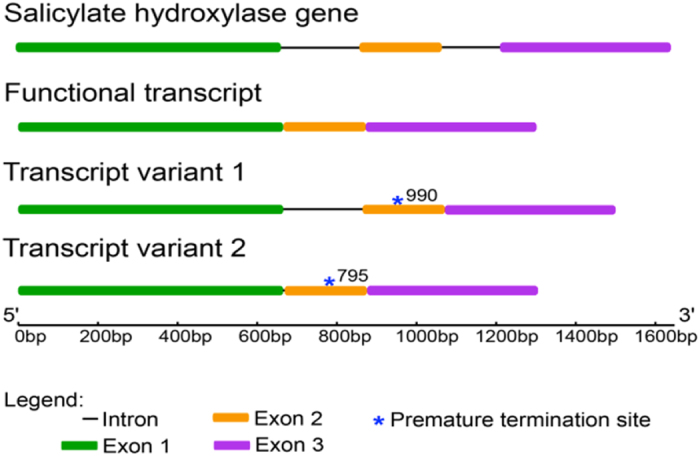
Gene structure of the *E. festucae* Rose City salicylate hydroxylase gene (accession KM400586), functional transcript, and alternatively spliced transcript variants. The exons are indicated by boxes and the introns by lines. The positions of the premature termination sites in the alternatively spliced transcript variants are indicated by asterisks.

*Efe-shyA* from the Rose City isolate of *E. festucae* was amplified and sequenced (NCBI accession KM400586). The sequence was identical to that in the genome of *E. festucae* 2368, EfM3.054130, (5; http://www.endophyte.uky.edu/) with the exception of one A to C SNP at position 1134 in the second intron. The gene is annotated as having two introns. A diagram of the gene structure is shown in Fig. 3.

**Figure 4:**
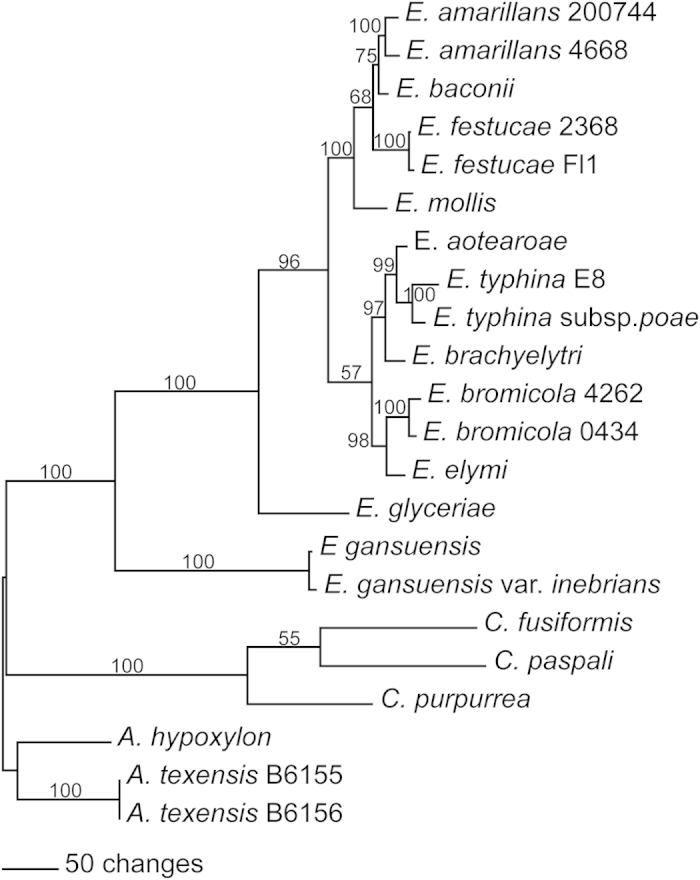
Rooted 50% majority rule maximum parsimony phylogenetic tree of *Epichloë* and related Clavicipitaceae spp. salicylate hydroxylase DNA coding sequences. The *Atkinsonella* spp. sequences were designated as the outgroup for rooting the tree. The numbers at the nodes are the bootstrap percentages based on 1000 replications. The tree was based upon 1438 total characters, of which 717 were constant, 213 variable characters were parsimony uninformative, and 580 variable characters were parsimony informative. Available NCBI accession numbers of the contigs containing the sequences are: *E. amarillans* 200744, AFRF01001066.1; *E. amarillans* 4668, JFGZ01000088.1; *E. baconii*, JFGY01000338.1; *E. festucae* 2368, ADFL02000346.1; *E. festucae* Fl1, AFRX01000200.1; *E. aotearoae*, JFGX01000019.1; *E. typhina* E8, AMDI01000203.1; *E. typhina* subsp. *poae*, AFSE01001014.1; *E. brachyelytri*, AFRB01001961.1; *E. elymi*, AMDJ01000092.1; *E. glyceriae*, AFRG01000890.1; *E. gansuensis*, AFRE01000003.1 and AFRE01000959.1; *E. gansuensis* var. *inebrians*, AMDK01001066.1.

Similar genes are present in all of the *Epichloë* spp. for which whole genome sequences are available, as well as some related Clavicipitaceae (http://www.endophyte.uky.edu/). A phylogenetic tree of the DNA coding sequences is shown in Fig. 4. Similar genes are also found in numerous additional fungal species for which whole genome sequences are available at NCBI.

**Figure 5:**
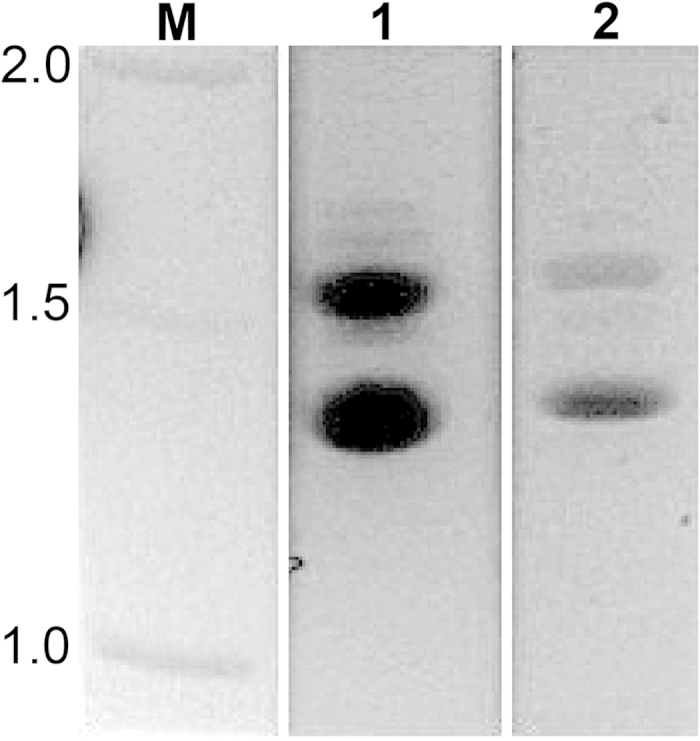
Expression of *E. festucae* Rose City salicylate hydroxylase *in vivo*. PCR product of the *E. festucae* Rose City salicylate hydroxylase transcript using oligo(dT)-generated cDNA reverse-transcribed from total RNA of (**1**) *E. festucae* Rose City fungal endophyte grown in culture, and (**2**) endophyte-infected *F. rubra* leaf sheath tissue. Standard DNA markers are indicated by M.

RT-PCR was used to evaluate expression of the gene in both *F. rubra* infected with the Rose City isolate of *E. festucae* and in the Rose City *E. festucae* fungal isolate grown in culture. Gel analysis of the PCR products revealed two bands in both samples indicating two potential transcripts for the gene (Fig. 5).

DNA sequencing analysis of the larger cDNA PCR product (1499 bp) revealed that the entire 199 bp of the first intron of the gene was retained in the coding sequence, indicating an alternatively spliced transcript variant (designed Transcript variant 1 in Fig. 3). This transcript variant would not be expected to produce a functional enzyme due to a premature termination codon at position 990. The smaller cDNA PCR product was also sequenced and found to be 1296 bp. The sequence of this transcript could be translated into a 431 amino acid protein of 47.48 kD, and was considered to be the functional salicylate hydroxylase (designated Functional transcript in Fig. 3).

The coding sequence of the putative functional transcript was subsequently cloned into the pET-21b(+) expression vector to determine the enzyme activity of the gene in *E. coli*. pET21b(+) introduces a C-terminal histidine tag on the protein and this is the same strategy that was used to determine the activity of NahG and NahU, another salicylate hydroxylase, from *Pseudomonas* sp. strain ND6^23^. Plasmids from the bacterial clones hosting the salicylate hydroxylase::pET21b(+) recombinant plasmid were sequenced to select for clones containing the correct 1296 bp salicylate hydroxylase sequence (described more below). The presence of a second alternatively spliced transcript of the *E. festucae* Rose City gene was detected at this stage (designated Transcript variant 2 in Fig. 3). This alternately spliced form of the gene included an extra 4 bp of the 199 bp first intron and resulted in a premature termination site at position 795 of the coding sequence. This transcript variant was also not expected to be translated into a functional protein.

### Catalytic Activity of the *Epichloë festucae* Salicylate Hydroxylase

**Figure 6:**
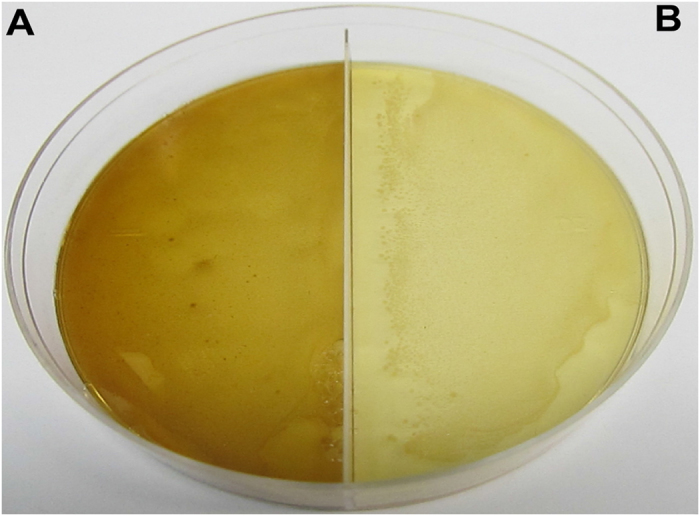
Colorimetric plate assay confirming the enzymatic acitivity of the *E. festucae* Rose City salicylate hydroxylase. The segmented LB plate was supplemented with 0.01 mM IPTG and 2.5 mM salicylic acid. (**A**) *E. coli* cells containing the pET21b(+) plasmid with the *E. festucae* Rose City salicylate hydroxylase. (**B**) *E. coli* cells containing the pET21b(+) vector only.

To assess the enzymatic acitivy of the *E. festucae* salicylate hydroxylase, the plasmid with the putative functional salicylate hydroxylase sequence from the smaller RT-PCR product described above was transformed into the *E. coli* BL21-CodonPlus (DE3)-RIPL expression strain. The first assessment of enzymatic activity was an *in vivo* plate assay in which 0.01 mM IPTG and 2.5 mM salicylic acid were incorporated into the bacterial medium in a divided Petri dish^24^. One side of the plate was innoculated with *E. coli* cells containing the pET21b(+) plasmid with the *E. festucae* Rose City salicylate hydroxylase and the other side was innoculated with *E. coli* cells containing the pET21b(+) vector only. Enzymatic activity can be visualized by the brown color produced from auto-oxidation of catechol, the product of salicylate hydroxylase activity (Fig. 6). These results confirmed that the cloned gene was indeed a salicylate hydroxylase.

**Figure 7:**
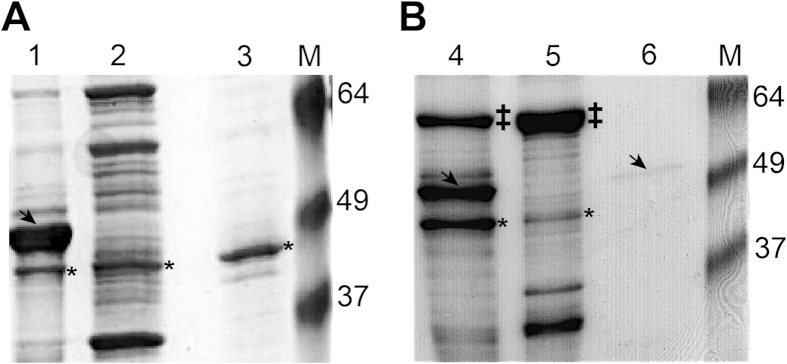
Expression of *E. festucae* Rose City salicylate hydroxylase in two *E. coli* expression strains. SDS-PAGE analysis of *E. festucae* Rose City salicylate hydroxylase expressed in *E. coli* (**A**) BL21 CodonPlus RIPL (DE) cells showing Lane 1: insoluble pellet, Lane 2: 25μg total soluble protein, and Lane 3: proteins eluted from nickel-ion magnetic beads consisting mainly of the contaminating 44 kD bacterial protein, (**B**) ArcticExpress RP (DE) cells showing Lane 4: insoluble pellet, Lane 5: 25 μg total soluble protein, and Lane 6: 0.5 μg purified protein using TALON Metal Affinity Resin. Arrow indicates presence of the induced protein at the expected size of 48.3 kD. Asterisk is used to show the nickel-binding contaminating *E. coli* protein. The 60 kD bacterial chaperonin protein, Cpn60 expressed in *E. coli* ArcticExpress cells is indicated by ‡. Standard protein markers are indicated by M.

Efforts to purify the *E. festucae* salicylate hydroxylase protein from the BL21-CodonPlus (DE3)-RIPL cells were unsuccessful. The bulk of the expressed protein was found in insoluble inclusion bodies (Fig. 7, lane 1). An approximately 44 kD *E. coli* protein out-competed the low level of soluble His-tagged protein when binding to Ni magnetic beads (Fig. 7, lane 3). The same Ni-binding protein was seen from lysates of untransformed *E. coli* cells (data not shown). Several metal binding *E. coli* proteins have been reported to be commonly co-purified by immobilised metal affinity chromatography, particularly when the amount of target protein is low^25^,^26^.

Soluble salicylate hydroxylase was obtained by expressing the protein in ArcticExpress RP(DE) bacterial cells and purification of the protein by using TALON Metal Affinity Resin (Fig. 7, lane 6). The ArcticExpress strain of *E. coli* was engineered to co-express the Cpn10 and Cpn60 cold-adapted chaperonins^27^ helping to increase the amount of soluble heterologous protein expression^28^,^29^,^30^. In contrast to the Ni magnetic beads, the cobalt-charged TALON Metal Affinity Resin did not co-purify the approximately 44 kD contaminating bacterial protein along with the target histidine-tagged recombinant salicylate hydroxylase protein. Similar purification results were reported for a 27 kD contaminating bacterial protein when using the TALON resin^31^. When expressed in ArcticExpress RP(DE) cells most of the *E. festucae* salicylate hydroxylase was still found in the insoluble fraction (Fig. 7, lane 4), but some soluble protein could be purified for enzymatic activity assays (Fig. 7, lane 6).

The activity of the purified recombinant protein was assayed against salicylic acid and 2,5-dihydroxybenzoic acid (gentisate). The enzyme had activity with both substrates (Table 1). The activity with gentisate was higher than with salicylic acid, as has been reported for other fungal and bacterial salicylate hydroxylase enzymes^11^,^32^.

### Salicylic Acid Levels in Endophyte-Free and Endophyte-Infected Plants

**Figure 8:**
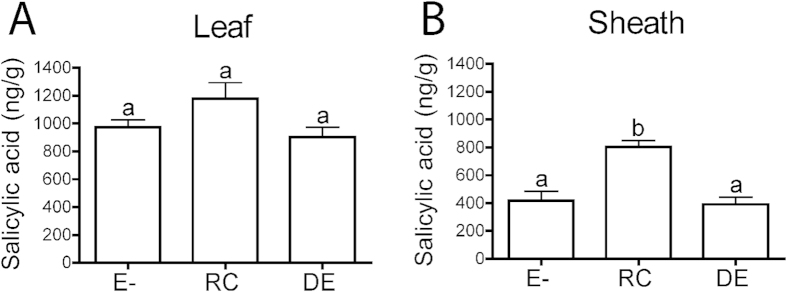
Salicylic acid levels in mature leaves and leaf sheaths of endophyte-free (S1139E-) and endophyte-infected (S1139Rose City and S1139Delaware) strong creeping red fescue plants. Samples with different letters are significantly different at *P* < 0.05.

Salicylic acid levels were determined in mature leaves and leaf sheaths of growth chamber grown endophyte-free (S1139E-) and endophyte-infected plants (S1139Rose City and S1139Delaware). The endophyte-infected plants are the same strong creeping red fescue plant genotype, S1139, that had been inoculated with either the Rose City or the Delaware isolates of *E. festucae*^33^. For all three plant samples, salicylic acid levels were higher in the leaves than in the leaf sheaths (Fig. 8). The salicylic acid levels in the leaves of endophyte-infected plants were not statistically different than in the leaves of the endophyte-free plant. The salicylic acid levels in S1139Rose City leaf sheaths were significantly higher than in the leaf sheaths of S1139E-. In contrast, the salicylic acid levels in the leaf sheaths of S1139Delaware were not significantly different than the levels in S1139E-. Overall these results do not support the hypothesis that expression of the endophyte salicylate hydroxylase may be a factor in the lack of a hypersensitive response by the host grass since the salicylic acid levels were not reduced in the endophyte-infected plants relative to the endophyte-free plant.

## Discussion

Here we report the first functional characterization of a fungal endophyte salicylate hydroxylase. The presence of the gene in the *E. festucae* genome was first recognized by expression of the fungal transcript *in planta*^7^. Numerous bacterial salicylate hydroxylase enzymes have been reported, with the most well known being NahG from *Pseudomonas putida*^8^. The only other fungal salicylate hydroxylase enzyme to be functionally characterized is Shy1 from the plant pathogen *Ustilago maydis*^15^. In that study, knockout of the *shy1* gene did not result in reduced virulence of the pathogen, indicating that fungal degradation of host salicylic acid is not a factor in virulence of *U. maydis*. The role of Shy1 in *U. maydis* is not yet known.

Although not functionally characterized, a genomic fragment from a terbinafine resistant isolate of *Aspergillus nidulans* containing a salicylate hydroxylase-like gene was able to confer terbinafine resistance to a susceptible isolate^12^. Terbinafine is an antifungal compound containing a naphthalene group. It was concluded that *A. nidulans* was catabolizing the naphthalene portion of terbinafine in a similar way as do bacteria, such as *Pseudomonas putida*, where salicylate hydroxylase is required to degrade the salicylic acid produced as an intermediate in naphthalene degradation. Similar salicylate hydroxylase-like genes, as well as other genes possibly involved in naphthalene degradation, are present in many fungal genomes, including saprophytes and pathogens^12^. The ability to degrade salicylic acid is apparently a common phenomenon among fungi. Recently an *Arabidopsis thaliana* salicylate hydroxylase was identified that converts salicylic acid to 2,3-dihydroxybenzoic acid, instead of catechol as do the bacterial and fungal enzymes^33^. The sequence of the plant enzyme is unrelated to the bacterial and fungal salicylate hydroxylases.

The *E. festucae* salicylate hydroxylase was previously considered to be a secreted protein based on bioinformatic prediction by the program TargetP^7^,^34^, but that is likely incorrect. The presence of the conserved N-terminal FAD-binding site in the predicted signal peptide region makes it unlikely that the N-terminus is a true signal peptide. A signal peptide is not predicted by the program SignalP^35^. The *U. maydis* salicylate hydroxylase Shy1 was shown to be cytoplasmically localized^15^.

In the SOLiD-SAGE analysis the *E. festucae* salicylate hydroxylase tag was identified as the 172nd most abundant fungal tag recovered from the infected host grass^7^. The tags are from the most 3’ *Nla*III fragments, and so would have included the non-functional transcript variant forms as well as the functional transcript. The tags for the functional transcript would be considerably less than previously thought since the level of transcript variant 1 was a large proportion of the total as can be seen in Fig. 5. Transcript variant 1 could be clearly seen as the larger RT-PCR band from cDNA isolated from *E. festucae* in culture as well as from the endophyte-infected plants. The relative proportions of transcript variant 2 and the functional transcript are not known since they are both present in the same smaller RT-PCR band. Both of the transcript variant forms contained portions of unspliced introns which resulted in a frameshift and a downstream early termination codon. The presence of unspliced introns in the salicylate hydroxylase transcript is not unique to the Rose City isolate of *E. festucae*. There are five ESTs (accession numbers GO805203, GO805370, GO814712, GO814910, GO851910) from an *E. festucae* isolate infecting meadow fescue (*Lolium pratense* = *Festuca pratensis*) and all are alternatively spliced variants of the gene. Apparently the sequence of the *E. festucae* salicylate hydroxylase gene makes it prone to generation of retained intron transcript variants. In a comparison of alternative splicing and splice recognition patterns across kingdoms, McGuire *et al*.^36^ reported that intron retention is more prominent among fungi and protists than in other eukaryotes and that intron retention was typically associated with weak splice site signals. They concluded that most transcript variants with retained introns are unlikely to result in a functional protein due to a shift in reading frame generating an early termination codon, consistent with the data presented here on the *E. festucae* salicylate hydroxylase.

Based on the comparison of salicylic acid levels between endophyte-free and endophyte-infected plants, it does not appear likely that expression of salicylate hydroxylase by the endophyte is a factor in the lack of a host defense reponse to the presence of the fungal endophyte. The physiological role of the *E. festucae* salicylate hydroxylase enzyme and how its expression impacts the endophyte-grass interaction are unknown. In the future, a knockout of *Efe-shyA* could be a useful approach in determining the role of salicylate hydroxylase in the symbiosis.

## Methods

### Plant and Fungal Materials

The strong creeping red fescue (*Festuca rubra* subsp. *rubra*) plants S1139E-, S1139Rose City, and S1139Delaware were described previously^37^. The endophyte-infected plant S1139Rose City was generated by inoculating an isolated tiller of the uninfected strong creeping red fesscue plant S1139E- with the Rose City isolate of *E. festucae*, which was isolated from an unrelated endophyte-infected strong creeping red fescue. The endophyte-infected plant S1139Delaware was generated by inoculating an isolated tiller of S1139E- with the Delaware isolate of *E. festucae*, which was isolated from a Chewings fescue (*F. rubra* subsp. *commutata*). Plants were clonally propagated and maintained in the greenhouse. These endophyte-infected plants have been stably maintained in the greenhouse for over 15 years.

The *E. festucae* Rose City isolate was isolated from the endophyte-infected S1139Rose City plant by plating surface-sterilized leaf sheath tissue on potato dextrose agar plates at room temperature (Difco Laboratories, Detroit, MI).

### DNA and RNA Isolation

Genomic DNA of *E. festucae* Rose City was extracted from culture grown in potato dextrose broth for 14 days on a shaker (175 rpm) at room temperature (23–25 °C). Fungal DNA was isolated using phenol-chloroform as previously described^38^.

The innermost leaf sheath tissue of endophyte-infected plant S1139Rose City was used for RNA isolation. For isolation of fungal RNA, the fungus was grown in potato dextrose broth for 14 days on a shaker (175 rpm) at room temperature. Each one g sample was ground to a fine powder with liquid nitrogen and resuspended in 10 mL Tri-Reagent (Sigma-Aldrich, St. Louis, MO). Debris was removed by centrifugation and the supernatant was extracted twice with chloroform. RNA in the aqueous layer was precipitated with 10 mL of isopropanol, and the RNA pellet was washed once with ethanol and dissolved in water.

Nucleic acid concentrations were measured by using a Nanodrop ND-1000 Spectrophotometer (Thermo Fisher Scientific, Waltham, MA).

### Amplification and Sequencing of *E. festucae* Rose City Salicylate Hydroxylase Gene and cDNA

First-strand cDNA of 5 μg *E. festucae* Rose City fungal or *E. festucae* Rose City-infected *Festuca rubra* plant total RNA was synthesized from 500 ng of oligo(dT)_18_ primer by using SuperScript™ III Reverse Transcriptase (Life Technologies, Carlsbad, CA) according to the manufacturer’s instructions.

PCR was performed in 50 μL reactions with either 1.0 μg of fungal genomic DNA, 1 μL *E. festucae* Rose City fungal cDNA, or 1 μL *E. festucae* Rose City-infected *Festuca rubra* plant cDNA, 1X PrimeSTAR Max Premix (Clontech Laboratories, Mountain View, CA), and 0.3 μM of each forward and reverse primer (Integrated DNA Technologies, Coralville, IA). Oligonucleotide primer sequences used for PCR amplification are those described below for cloning of the gene in *Escherichia coli*. Forward and reverse primers were designed based on the *E. festucae* 2368 genome sequence, EfM3.054130, (5; http://www.endophyte.uky.edu/). PCR was done in a GeneAmp 9700 thermocycler (Applied Biosystems, Inc., Foster City, CA) with 35 cycles of denaturation at 98 °C for 10 s, followed by 30 s annealing at 55 °C, and 30 s extension at 72 °C. The PCR products were visualized on a 1% TBE agarose gel. The two bands were each excised from the gel and purified by using a commercial kit (Qiaquick Gel Extraction Kit, Qiagen, Valencia, CA). In order to obtain enough DNA for sequencing and cloning, the DNA recovered from each band was used as the template in a second PCR reaction.

Each PCR product from the second reaction was sequenced directly (Genewiz, Inc., South Plainfield, NJ) after purification using 0.5X Agencourt AMPure XP (Beckman Coulter, Brea, CA) to remove any fragments under 1 kb. For each sequencing reaction, 40 ng of purified PCR product in 10 μL was treated with 2 μL ExoSAP-IT (Affymetrix, Santa Clara, CA) to remove unincorporated primers and excess dNTPs. The ExoSAP-IT reaction was performed at 37 °C for 15 min followed by heating at 80 °C for 15 min to inactivate the enzymes. Sequencing was done in both directions. The salicylate hydroxylase gene in the *E. festucae* Rose City isolate was also amplified and sequenced to confirm intron lengths and positions.

### Cloning of the *E. festucae* Rose City Salicylate Hydroxylase Gene in *Escherichia coli*

To test the activity of the *E. festucae* Rose City salicylate hydroxylase enzyme, the PCR product from the smaller band described above was cloned into the expression vector pET-21b(+) (Novagen, Billerica, MA), which introduces a C-terminal histidine tag. The *E. festucae* Rose City cDNA was amplified by PCR as described above with oligonucleotides that introduced a *Nde*I site at the 5’ end and a *Xho*I site at the 3’ end. The sequences of the oligonucleotides are: forward 5’- GATATACATATGGCGACCAAGAAAGACCA-3’ and reverse 5’-TCAACTCTCGAGACCAACGGCACGCCACCT -3’. Restriction enzyme sequences are underlined. Two hundred ng of the purified PCR product were digested with the restriction enzyme *Nde*I (TaKaRa Bio, Shiga, Japan), purified with 0.5X Agencourt AMPure XP to remove any fragments under 1.0 kb, digested with *Xho*I, and again purified with 0.5X AMPure XP. The expression vector pET21b(+) was similarly digested with *Nde*I and *Xho*I. Overnight ligation of the digested *E. festucae* Rose City salicylate hydroxylase PCR fragment and pET21b(+) plasmid was done at a 2:1 insert:vector molar ratio using T4 DNA ligase (New England Biolabs, Ipswich, MA).

Two μL of the ligation product was used to transform 20 μL *E. coli* DH5α electroporation-competent cells. The transformed cells were incubated in SOC medium for 1 hour at 37 °C with shaking, followed by overnight growth of cells on LB medium supplemented with 100 μg mL^−1^ ampicillin. Transformed bacterial colonies were screened for recombinant plasmids containing the *E. festucae* Rose City salicylate hydroxylase gene insert by using PCR as described above. Selected clones were grown overnight in 5 mL LB supplemented with 100 μg mL^−1^ ampicillin at 37 °C on a shaker (200 rpm), followed by plasmid purification using QIAprep Spin Miniprep Kit (Qiagen). DNA sequencing of the plasmids was done as described above. The inserts from two clones were sequenced and one had the correct sequence. The other retained a 4 bp region of intron one, and was designated Transcript variant 2. The plasmid containing the correct sequence was transformed into two *E. coli* competent cells strains; these were BL21-CodonPlus (DE)-RIPL (Agilent Technologies, Santa Clara, CA) and ArcticExpress RP (DE) (Clontech Laboratories). Transformants containing pET21b(+) vector were only generated for use as negative controls.

### Expression of the *E. festucae* Rose City Salicylate Hydroxylase Protein in *E. coli*

To confirm expression of the cloned salicylate hydroxylase gene, *E. coli* BL21-CodonPlus (DE3)-RIPL transformant cells containing the *E. festucae* Rose City salicylate hydroxylase::pET21b(+) recombinant plasmid were grown overnight in LB supplemented with 100 μg mL^−1^ ampicillin at 37 °C on a shaker (200 rpm). Cells were plated onto LB medium containing 100 μg mL^−1^ ampicillin, 2.5 mM salicylic acid and 0.01 mM isopropyl-β-D-1-thiogalactopyranoside (IPTG) on one half of a segmented Petri dish. *E. coli* cells carrying the pET21b(+) vector only were plated on the other half of the Petri dish as a negative control. Plates were examined for accumulation of dark brown auto-oxidation products of catechol, the product of salicylate hydroxylase activity^24^.

For attempted purification of the *E. festucae* Rose City salicylate hydroxylase protein in BL21-CodonPlus (DE3)-RIPL, transformant cells were grown overnight in LB and 100 μg mL^−1^ ampicillin at 37 °C. A 1:20 dilution of the overnight culture was added to fresh LB supplemented with 100 μg/mL ampicillin and incubated at 37 °C on a shaker until OD 0.5-0.6. To induce protein expression, IPTG was added to 0. 01 mM to the culture and then incubated at 20 °C with rotational shaking for 4 hrs. *E. coli* cells expressing the recombinant protein were pelleted and lysed using 1X FastBreak Cell Lysis Reagent (Promega, Madison, WI), 0.1 mg mL^−1^ DNaseI (Sigma-Aldrich), 200 μg mL^−1^ lysozyme (Sigma-Aldrich) and 1X Protease Inhibitor Cocktail (Sigma-Aldrich). Following lysis, samples were centrifuged at 10,000 rpm for 15 min and the supernatant removed. The soluble phase was treated with MagneHis Protein Purification System (Promega) to bind and purify the 6X Histidine-tagged recombinant protein following the manufacturer’s protocol.

The pET21b(+) plasmid containing the *E. festucae* Rose City salicylate hydroxylase coding sequence was also transformed into ArcticExpress RP (DE) cells to improve protein solubility. A 0.5 L bacterial culture was grown in LB plus 100 μg mL^−1^ carbenicillin and 20 μg mL^−1^ gentamycin to an OD600 of 0.525. To induce protein expression, IPTG was added to a final concentration of 0.01 mM. The culture was then grown for 21 hrs at 10 °C with rotational shaking. ArcticExpress RP (DE) bacterial cells expressing the salicylate hydroxylase protein were pelleted and lysed using 108 mL xTractor Cell Lysis Buffer (Clontech Laboratories), 0.1 mg mL^−1^ DNaseI (Sigma-Aldrich), 200 μg mL^−1^ lysozyme (Sigma-Aldrich) and 1X Protease Inhibitor Cocktail (Sigma-Aldrich). Following lysis, samples were centrifuged at 10,000 rpm for 30 min and the supernatant removed. The soluble phase was treated with 1 mL TALON Metal Affinity Resin (Clontech Laboratories) to purify the His-tagged protein following the manufacturer’s protocol. Imidazole was removed from the purified protein sample by using a PD-10 desalting column (GE Healthcare Life Sciences, Pittsburgh, PA) equilibrated with 33 mM potassium phosphate buffer, pH 7. The sample was concentrated using an Amicon Ultra 4 mL centrifugal filter with a 10 kD molecular weight cut-off (EMD Millipore Corporation, Billerica, MA). The concentrated purified protein was quantified using Bio-Rad Protein Assay Dye Reagent Concentrate (Bio-Rad Laboratories, Hercules, CA).

For sodium dodecyl sulfate-polyacrylamide gel electrophoresis (SDS-PAGE) analysis, protein samples were mixed with an equal volume of 2x SDS sample buffer [2x; 125 mM Tris, pH 6.8, 4.6% glycerol, and 0.002% bromophenol blue (w/v)^39^, then boiled for 5 min and subjected to electrophoresis on 10% polyacrylamide gels. Gels were stained with Coomassie Blue to visualize protein bands. Gels were destained, and then dried using a modified protocol from Moghaddam and Reinton^40^. Gels were soaked in a solution of 50% (v/v) methanol and 20% (v/v) polyethylene glycol 400 (PEG-400). Cellophane sheets, first soaked in water, were used to sandwich the gels in a gel-drying frame. The gels were dried at room temperature overnight.

### Enzyme Activity Assays of *E. festucae* Rose City Salicylate Hydroxylase

The salicylate hydroxylase enzyme assays were as described by Zhao *et al*.^23^. Half a microgram of purified recombinant salicylate hydroxylase was used per reaction. Each reaction contained 150 μM of salicylic acid or 2-5-dihydroxybenzoic acid, 120 μM NADH (Sigma-Aldrich), 10 μM FAD (Sigma-Aldrich), and 33 mM potassium phosphate buffer, pH 7 in a final volume of 1 mL. The reactions were assayed in triplicate. Oxidation of NADH was monitored as a decrease of absorbance at 340 nm (ε_NADH_ = 6200 M^−1^ cm^−1^) by using a UV160-U UV-VIS spectrophotometer (Shimadzu, Kyoto, Japan). Specific activities were expressed as nmol NADH min^−1^ mg protein^−1^.

### Salicylic Acid Measurement

Mature leaves and leaf sheaths (4-5 replicates) were collected from S1139E-, S1139Rose City and S1139Delaware growth chamber grown plants. The growth chamber conditions were set to 22/17 °C (day/night) temperature, 60% relative humidity, 650 μmol•m^−2^•s^−1^ photosynthetic active radiation and a 14-h photoperiod. The tissues were ground with liquid nitrogen into a powder. The powder (0.5 g) was extracted with 80% acetone twice (1.4 mL for the first extraction and 0.6 mL for the second extraction), vortexed thoroughly and sonicated for 20 min. Following centrifugation at 10,000 g for 20 min, the supernatants were combined. A recovery control sample containing 2 mL 80% acetone and 1 μg salicylic acid was also prepared. All samples were dried in an exhaust hood overnight and re-suspended in 0.2 mL 5 mM sodium acetate buffer containing 80 units g^−1^ β-glucosidase, pH 5.5^41^,^42^. The samples were incubated at 30 °C in a water bath for 90 min, then mixed with 0.8 mL 5% trichloroacetic acid and centrifuged at 10,000 g for 15 min. The supernatants were collected and extracted with 0.5 mL extraction buffer (ethylacetate/cyclopentane/isopropanol, 100/99/1, v/v/v) 3 times by vortexing and centrifuging at 10,000 g for 1 min. All upper phases were combined and condensed in an exhaust hood overnight. The almost dry samples were resuspended in 0.25 mL 100% methanol, vortexed thoroughly, sonicated and centrifuged for 5 min each.

The supernatants were analyzed by using a Waters HPLC system comprised of a Waters 600 Controller, an in-line degasser, a 717 plus autosampler, a SunFire^TM^ C18 (250 × 4.6 mm, 5 μm) reverse phase column, and a 996 photodiode array detector. Separations were carried out in a binary solvent system: solvent A was 0.1% formic acid in water, and solvent B was 0.1% formic acid in acetonitrile. A linear gradient of 0–20% B from 0 to 5 min; 20–30% B from 5 to 20 min; 30–32% B from 20 to 25 min; 32–45% B from 25 to 27 min; an isocratic elution with 45% B from 27 to 32 min; a linear gradient of 45–90% B from 32 to 35 min; an isocratic elution with 90% B from 35 to 38 min; a linear gradient of 90–100% B from 38 to 40 min; 100-0% B from 40 to 45 min; and an isocratic elution with 0% B from 45 to 55 min. Salicylic acid was eluted after 32 min with a flow rate of 1 mL min^−1^. The detector detection wavelength was 302 nm. The salicylic acid extraction efficiency was 50%.

### Phylogenetic Analysis

The salicylate hydroxylase DNA coding sequences were obtained from the Genome Project at the University of Kentucky website (5; http://www.endophyte.uky.edu/). The Clustal-X program^43^ was used to align the sequences. The alignments generated by Clustal-X were modified manually to minimize gaps. The phylogenetic analysis was performed with the PAUP^*^ program, version 4.0b10 for Macintosh. The phylogenetic analysis was done by using the maximum parsimony full heuristic search option set to random sequence addition, tree-bisection-reconnection (TBR) branch swapping, and Multrees on, with 1000 bootstrap replications. Gaps were treated as missing data.

### Schematic Representation of *Efe-shyA* gene structure

Schematic representation of the *E. festucae* Rose City salicylate hydroxylase gene and alternatively spliced transcript variants was done using Gene Structure Display Server^44^; (http://gsds.cbi.pku.edu.cn/index.php?input=site).

## Additional Information

**How to cite this article**: Ambrose, K. V. *et al*. Functional characterization of salicylate hydroxylase from the fungal endophyte *Epichloë festucae*. *Sci. Rep*. **5**, 10939; doi: 10.1038/srep10939 (2015).

## Figures and Tables

**Figure 1:**
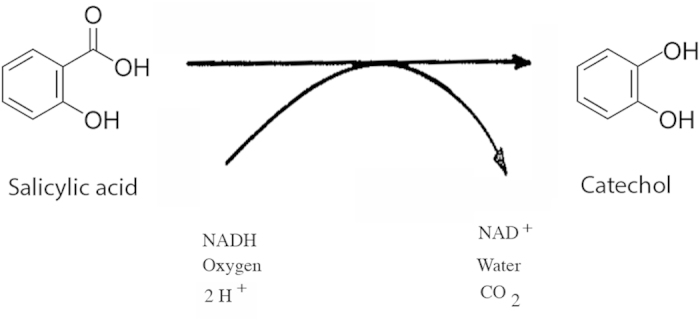
Reaction catalyzed by salicylate hydroxylase (E.C.1.14.12.1).

**Table 1:** Enzymatic activity of the purified recombinant *E. festucae* Rose City salicylate hydroxylase.

Substrate	Activity	Relative activity (%)
Salicylate	713 ± 75	100
Gentisate	1038 ± 68	146

Activities are the means of triplicate assays ± SD and are presented in nanomoles NADH minute^−1^ milligram protein^−1^.
